# Building a Virtual Community of Practice for Family and Friend Caregivers of People Living With Dementia: A Mixed-Methods Study

**DOI:** 10.1177/23779608261473148

**Published:** 2026-07-23

**Authors:** Marie-Lee Yous, Ruthie Zhuang, Nicole Beaudry, Susanne Langdon, Paul Lee, Myrna Norman, Sylvie Sylvestre, Aki Tomizawa, Sharon Kaasalainen, Carrie McAiney

**Affiliations:** 1 School of Nursing, 63661 McMaster University, Hamilton, ON, Canada; 2 Michael G. DeGroote School of Medicine, 63661 McMaster University, Hamilton, ON, Canada; 3 School of Public Health Sciences, Schlegel-UW Research Institute for Aging, 8430 Schlegel Research Chair in Dementia University of Waterloo, Waterloo, ON, Canada

**Keywords:** dementia, family caregiving, caregivers, Namaste care, virtual

## Abstract

**Introduction:**

Over 55 million people worldwide live with dementia, placing significant demands on family and friend caregivers. These demands lead to stress, depression, and social isolation. Caregivers report unmet needs such as access to resources, education, and emotional support. A Virtual Community of Practice (VCoP) offers a promising solution by fostering shared learning, emotional support, and practical caregiving strategies.

**Objectives:**

This study explored the (a) perceptions of caregivers and a person living with dementia of the VCoP (b) recommendations of caregivers and a person living with dementia to improve the VCoP and (c) experiences of caregivers and a person living with dementia in co-designing the VCoP.

**Methods:**

Using a mixed-method sequential explanatory approach, ten research partners (nine caregivers and one person with dementia) participated in two virtual workshops and follow-up interviews. Survey data were collected via the System Usability Scale (SUS) and Patient and Public Engagement Evaluation Tool (PPEET). Survey data were analyzed using descriptive statistics. Thematic analysis of workshop and interview data was conducted.

**Results:**

The VCoP achieved an SUS score of 94.5, indicating excellent usability. PPEET results showed high satisfaction with engagement in development. A total of eight themes were developed. Thematic analysis highlighted strengths such as ease of locating information on the website, practical content, and opportunities for sharing and learning through discussion forums. Recommendations included improving accessibility, tailoring activities to life stories, and ensuring activities maintain normalcy. Research partners felt included and supported during the workshops, enjoyed the small group atmosphere, and appreciated seeing their ideas reflected in the final product.

**Conclusions:**

The VCoP was perceived as practical for supporting caregivers. Involving people with lived experience ensured the intervention met caregiver needs. Findings underscore the importance of VCoPs and the value of co-design in creating effective caregiver support tools.

## Introduction

Dementia affects over 55 million people globally (Alzheimer Disease International, 2025), with 733,040 individuals currently living with dementia in Canada and prevalence expected to increase by 65% between 2020 and 2030 (Alzheimer Society of Canada, 2025). Dementia includes symptoms such as memory loss and impaired cognition associated with neurodegenerative diseases such as Alzheimer’s disease, which significantly impair daily functioning (Alzheimer’s Association, 2025) and create care demands that exceed those of many chronic conditions (Reuben et al., 2022). Family and friend caregivers of individuals with dementia provide an average of 26 hours of care per week compared to 17 hours for other conditions (Alzheimer Society of Canada, 2025). In this paper, the term ‘caregiver’ will be used to describe informal, unpaid, and family/friend caregivers.

Care needs often include support with activities of daily living (ADLs), behavioral symptom management, coordination of comorbidities, and meaningful engagement (Alzheimer’s Association, 2025; Reuben et al., 2022). Most individuals (61%) live in the community, with many requiring extensive assistance (Canadian Institute for Health Information, 2025). Family caregivers remain involved even in long-term care (LTC), providing emotional, social, and physical support through regular visits (Boamah et al., 2024; Weimer et al., 2022).

Caregiving responsibilities are time-intensive and emotionally demanding, particularly for individuals balancing work and family roles (Smith et al., 2022). These demands contribute to caregiver stress, depression, financial burden, and social isolation (Sorrentino et al., 2025), while social support can mitigate these effects (Nemcikova et al., 2023). Despite this, unmet needs remain high, with many caregivers lacking access to support groups, education, and resources (Sorrentino et al., 2025; Zwingmann et al., 2019). Addressing these gaps is essential to improving quality of life for caregivers and persons living with dementia.

## Review of Literature

Communities of Practice (CoPs) are a promising strategy for supporting caregivers through shared learning, knowledge exchange, and peer support (Rogers, 2000; Wenger, 1998). They facilitate collaborative learning and have been shown to improve health education, clinical outcomes, and social connection (Noar et al., 2023; Romero-Mas et al., 2024). Virtual Communities of Practices (VCoPs) expand accessibility and flexibility, enabling participation among caregivers facing high care demands or limited access to in-person supports (Dedzoe et al., 2023).

VCoPs also support delivery of psychosocial interventions that improve caregiver well-being and confidence while enhancing outcomes for people living with dementia (Simard and Press, 2023; Tang & Chan, 2016; Wiegelmann et al., 2021; Yous et al., 2023). One such intervention is Namaste Care, a multisensory program designed to enhance quality of life through individualized, calming activities (Simard and Press, 2023). Evidence suggests Namaste Care reduces pain, psychotropic medication use, and responsive behaviors while improving relationships between caregivers and care recipients (McNiel & Westphal, 2018; Smaling et al., 2025; Yous et al., 2022).

Providing structured education and support is essential for effective dementia care (Yous et al., 2022). However, many digital interventions are developed without meaningful input from caregivers or persons living with dementia, limiting relevance and usability (Kirby et al., 2024). Co-design approaches have shown improved engagement and reduced caregiver distress (Gitlin et al., 2022), yet few VCoPs incorporate interest-holder involvement (Romero-Mas et al., 2021; Romero-Mas et al., 2024). One important educational need addressed in VCoPs is supporting caregivers in delivering meaningful activities, including Namaste Care. Caregivers may benefit from training and access to tools that facilitate engagement and enhance care experiences. We were also interested in exploring the experiences of research partners in the co-design process to determine whether they contributed to their full ability, and their ideas were incorporated to their satisfaction. The purpose of this study was to examine the perceptions and experiences of caregivers and a person living with dementia participating in and co-designing a VCoP, and to gather their recommendations for enhancing the VCoP.

## Methods

### Intervention

Research partners contributed to the design, layout, and resources on the VCoP website and provided feedback on how Namaste Care would be delivered. The VCoP included the following four components: (a) a user-friendly publicly available website (https://www.vcopforcaregivers.com), (b) online resources (e.g., toolkit, video clips, evidence-based dementia care strategies) related to topics such as dementia knowledge, pain management, nutrition, and financial resources,, (c) virtual training and support by a Registered Nurse for caregivers to deliver Namaste Care, and (d) 60-minute virtual discussion forums for caregivers hosted by a Registered Nurse occurring every two weeks to discuss the delivery of Namaste Care and a topic related to dementia caregiving (e.g., pain management, decision-making, self-care, nutrition, palliative care, medications). The website includes information and online resources only. Caregivers seeking virtual training to deliver Namaste Care and wanting to be part of bi-weekly discussion forums were provided with a secure Zoom link. Content and the format of bi-weekly virtual discussion forums (e.g., length, topics, structure) were determined by research partners.

### Research Design

This study employed a mixed methods sequential explanatory design, in which quantitative data were collected and analyzed first, followed by qualitative data collection and analysis to explain and contextualize the quantitative results (Creswell & Plano Clark, 2018). Quantitative data were obtained through the System Usability Scale (SUS) and the Patient and Public Engagement Evaluation Tool (PPEET) (Abelson et al., 2016; Brooke, 1996) and analyzed descriptively to assess participants' perceptions of usability and engagement with the VCoP. These findings informed the subsequent qualitative phase, which consisted of workshops and interviews aimed at exploring participants' experiences and providing greater insight into patterns identified in the survey data.

A qualitative descriptive approach was used to produce a rich yet straightforward account of participants' experiences and perspectives, remaining close to the data while allowing for interpretive insight (Sandelowski, 2000; Sandelowski, 2010). This approach facilitated a deeper understanding of how caregivers interacted with the VCoP and how participant feedback could be incorporated to better align the intervention with caregiver needs. Methodological rigor was guided by the Mixed Methods Appraisal Tool (MMAT) (see supplementary file).

Integration occurred during interpretation, where qualitative findings were used to explain, elaborate upon, and contextualize the quantitative results. For example, high SUS scores were further illuminated by participants' descriptions of intuitive navigation, ease of use, and accessibility. Similarly, positive PPEET scores were explained through qualitative accounts of meaningful participation, inclusivity, and a sense of community. The integration of quantitative and qualitative findings provided a more comprehensive understanding of caregiver experiences and strengthened the overall validity and depth of the study.

### Research Questions

The research questions of this current study were: (a) What are the perceptions of caregivers and a person living with dementia of the VCoP (b) What are the recommendations of caregivers and a person living with dementia to improve the VCoP and (c) What were the experiences of caregivers and a person living with dementia in co-designing the VCoP?

### Setting

The research partners attended online workshops through an encrypted videoconferencing platform (i.e., Zoom) where they shared their insights and feedback on their ideas for a VCoP. Support in the form of technological devices, internet access, training in the videoconferencing software and reimbursement for respite for active caregivers was offered to all research partners. Upon completing the workshop sessions and within two weeks, individual follow-up interviews were conducted through either video or teleconference to assess the research partners’ perceptions of their engagement throughout the workshops.

### Sample and Inclusion/Exclusion Criteria

The sample consisted of one person with dementia and nine family/friend caregivers of people living with dementia. Persons with dementia were asked to self-disclose a diagnosis of dementia and have the capacity to complete surveys and participate in study activities independently or with the assistance of a family member or friend. We relied on their self-assessment to determine whether they could take part in the study. Eligible participants include adults living in Canada who can communicate in English and provide informed consent, comprising (1) individuals aged 40 years or older diagnosed with dementia, and (2) adults aged 18 years or older with experience within the past five years providing physical, emotional, and/or psychological support to a family member or friend with dementia in a home or LTC setting. Caregivers were defined as unpaid caregivers providing support or physical care for a relative with dementia for at least 4 hours a week. Caregivers who were no longer caring for their loved one due to their passing were included. This study inclusion criteria for persons living with dementia and caregivers are listed in Table 1. Lastly, maximum variation sampling was used to seek diverse research partners in terms of sex, ethnic background, and level of education. The only exclusion criterion was that research partners had to be able to converse in English. Sample size was determined based on previous studies exploring co-design including 6-13 participants (McGowan et al., 2024; Pallesen et al., 2020; Yous et al., 2022).

**Table 1.** Study Inclusion Criteria

Research partner group	Criteria
Persons living with dementia	(a) aged 40 years or older with a diagnosis of dementia, (b) able to speak, write, and understand English, (c) currently living in Canada, and (d) able to provide informed consent.
Caregiver	(a) aged 18 years or older and with experience in the last five years providing physical, emotional, and/or psychological support for a family member or friend with dementia in a home or LTC setting, (b) able to speak, write, and understand English, (c) currently living in Canada, and (d) able to provide informed consent.

### Recruitment

Research partners were recruited from organizations across Canada that target or provide services for older adults, caregivers (i.e., family members or friends), and/or people living with dementia including the Alzheimer Society of Canada and the Engagement of People with Lived Experience of Dementia (EPLED) group. Recruitment occurred through presentations at dementia education events for persons with dementia and caregivers and electronic recruitment posters shared through the Alzheimer Society. Additionally, snowball sampling was used as research partners were asked to share the study and sign-up information with others who may be interested in participating.

## Data Collection

Data were collected from January to August of 2023. Demographic characteristics of research partners such as sex and age were collected using questionnaires at recruitment. Research partners completed the SUS to evaluate the usability of VCoP website and the PPEET in evaluating the quality of the engagement of research partners in the workshop sessions (Abelson et al., 2016; Brooke, 1996). SUS consists of a scale of 1 to 5 indicating agreement with a total score ranging from 0-100 with higher scores representing more favorable results A score of greater than 80.3 indicates “excellent” or grade of A, a score of 68-80.3 indicates “good” or grade of B, a score of 68 means “okay” or grade of C, a score of 51-68 means “poor” or grade of D, and a score of less than 51 indicates “awful” or grade of F (Brooke, 1996). The PPEET tool consists of an assessment tool with narrative options which does not provide an overall score (Abelson et al., 2016). Surveys were completed electronically using a secure link approximately one to two weeks following completion of the second workshop discussion session.

Qualitative data were collected through videoconference workshop discussion sessions with research partners and this was led by the first author with experience in co-design (MY). Virtual semi-structured interviews lasting approximately 30 minutes were conducted approximately one to two weeks after the second workshop was completed with each research partner by a research assistant with training in conducting interviews (RZ). This follow-up interview explored the research partner’s engagement experiences. The interview guide was based on the goals of the Strategy for Patient-Oriented Research (SPOR) patient engagement framework: (a) inclusive mechanisms and processes created, (b) respectful collaboration between researchers and research partners, and (c) experiential knowledge of research partners is valued as evidence for research (Canadian Institutes for Health Research, 2014). Both workshop sessions and interviews were audio-recorded and transcribed by an experienced transcriptionist. Data collection continued until data saturation was reached.

### Workshops

The development of the VCoP was based on the key elements of Wenger’s (1998) framework for CoP, being: (1) joint enterprise (collective understanding of the VCoP’s purpose), (2) mutual engagement (interacting and creating expectations, norms and relationships), and (3) shared repertoire (using communal resources) (Wenger, 1998). Ultimately, we sought to bring together the member’s shared goals and imaginations to create an effective VCoP for dementia caregivers.

In total, each research partner attended two 60-minute virtual small group workshop sessions facilitated by MY with typically three research partners attending each. Additionally, each research partner met individually with the facilitator virtually or via teleconference to help prepare for the first workshop and address any questions. Research partners were offered access to a tablet if they did not have one and a training session in how to join Zoom meetings.

In the first session, research partners were asked to review the draft VCoP website with preliminary content related to dementia and caregiving (e.g., information on Namaste Care, links to the Alzheimer’s Society of Canada) and provide recommendations to improve the site based on the content, resources, language used, layout, and ease of navigation. They were asked to provide suggestions on what kinds of demonstrative video clips and information would be helpful to support the delivery of social and engaging activities for persons with dementia. Research partners were also asked to provide insights on the length of time, number of people per group, and relevant topics for the online discussion forums. All research partners were offered opportunities to be an author on manuscripts and presenters at conferences/meetings.

Research partners listened to a 15-minute presentation describing what a VCoP was, the Namaste Care program, and the purpose of their collaboration. A rough draft of the website was presented to the research partners as a starting point for discussion. Additionally, the facilitator explained how to use the types of products found in a sample Namaste Care Toolbox with the support of photos and YouTube videos. Following the presentation, the research partners participated in workshop discussions for 45 minutes where they shared ideas about building the VCoP and resources to include on the website.

Prior to the second workshop session, research partners were emailed links and drafts of materials to review before the session. At the second session, a 20-minute presentation of the draft VCoP website was presented and partners were asked whether they felt their feedback from the last workshop had been incorporated and if any other changes were needed. The Canadian Institutes of Health Research’s SPOR Patient Engagement Framework was used to guide workshop discussions and support meaningful involvement of participants as research partners (Canadian Institutes of Health Research, 2014). The framework emphasizes inclusiveness, support, mutual respect, and co-building and informed the design and facilitation of workshop activities. Questions were developed to reflect these principles by valuing lived experience and promoting collaboration. For example, participants were asked about their caregiving experiences and perspectives on workshop content and design. This approach ensured active engagement and supported the integration of participant input throughout the research process (Canadian Institutes of Health Research, 2014). For example, in terms of inclusiveness and valuing lived experience, questions such as “What are your experiences in being a family or friend caregiver?” and “How have you adapted to changes brought on by dementia?” were included.

### Evaluation of Patient Engagement

A research assistant (RZ) conducted short qualitative interviews virtually by Zoom or phone post-workshop session lasting 20-45 minutes using the Patient and Public Engagement Evaluation Tool (PPEET) to capture experiences at the workshop sessions and determine areas of strength and improvement (Abelson et al., 2016).

### Data Analysis

Quantitative data were analyzed descriptively as categories, counts, means, and standard deviations. Qualitative data obtained from the workshop sessions and interviews were analyzed separately using experiential thematic analysis, which focuses on the experiences of participants and their understanding of their world (Braun & Clarke, 2022). The data were analyzed using the six phases of thematic analysis as outlined by Braun and Clarke (Braun & Clarke, 2022): (a) gaining familiarity with the data, (b) conducting coding, (c) locating themes, (d) reviewing themes, (e) developing a definition for themes and naming them, and (f) developing a report.

Data were reviewed twice by the first author (MY) and a research assistant (RZ) before beginning to code, and initial reactions to the data were documented. Data were analyzed concurrently as interviews were being completed. Additionally, feedback from co-investigators ensured that the coding process was sound. Constant comparative analysis was used to identify similarities and differences across participants and participant groups (Sandelowski, 2000). MY and RZ reviewed themes and resolved conflicts when they arose through regular meetings. Themes were developed from the codes to highlight meaningful patterns within the data while reflecting the research questions. Inductive, data-driven coding was used to remain close to participants words and avoid trying to fit data into an existing framework, aligning with qualitative descriptive design (Sandelowski, 2000). Co-investigators had expertise in co-design, dementia research, and implementation and evaluation of interventions for caregivers. NVivo version 2020 software was used for data management.

Mixed methods data analysis followed a sequential explanatory design (Creswell & Plano Clark, 2018). Quantitative data (SUS and PPEET) were analyzed first using descriptive statistics. Qualitative workshop and interview data were then analyzed using thematic analysis to explain and expand upon the quantitative findings. Integration occurred during interpretation, where qualitative themes provided context for quantitative results.

### Ethical Considerations

Ethics approval was received from the Hamilton Integrated Research Ethics Board (#14930). The three core principles, respect for persons, concerns for welfare and justice, of the Tri-Council Policy Statement were applied throughout the study (Government of Canada, 2019). All research partners received a written introduction to the study and an informed consent form written in lay language. Lastly, after the workshop and follow-up interviews were completed, research partners were thanked for their time and contributions. A $100 gift card of their choice was provided as a token of appreciation.

## Results

### Demographic Results

Demographic data was grouped together for the total of 10 research partners to avoid being able to potentially identify the single research partner who shared that they were living with dementia. The mean age of research partners was 61.1 years with a standard deviation (SD) of 13.4. The person living with dementia was 74 years old at the time of the study. All but one research partner identified as being female. There was diverse representation with regards to race and ethnicity. The years of experience that caregivers had in supporting a family member varied with a mean of 11.7 years (SD = 14.7). See Table 2 for the demographic characteristics.

**Table 2.** Research Partners Demographic Characteristics (N=10)

Category	n
Age in years (Mean [SD])	61.1 [13.4]
Sex	
Male	1
Female	9
Race/ethnicity (May select up to 4)	
White	6
Indigenous	1
Chinese	1
Japanese	1
Filipino	1
Black	1
Relationship to person with dementia	
Spouse	1
Son/daughter	8
Receiving support as a person living with dementia from:	
Spouse	1
Number of years as a caregiver (Mean [SD])	11.7 [14.7]
Number of years living with dementia (if applicable)	13 years

SD = Standard Deviation.

### System of Usability Scale and Patient and Public Engagement Evaluation Tool Results

With regards to the usability of the VCoP, the survey results revealed that most partners found the website and its resources to be easy to access and helpful for caregivers. The VCoP website had a total score of 94.5 indicating a “excellent” or grade of A. See Table 3 for a summary of results for each question of the SUS (Brooke, 1996). The PPEET results were similarly favorable (Abelson et al., 2016). Most research partners felt that they had a clear understanding of the purpose of the workshop and enough information to contribute to discussions. All partners reported they were able to express their views freely, their views were heard, satisfaction with the workshops, and that the workshops were a good use of their time. See the supplementary file for a summary of results for each question of the PPEET.

**Table 3.** System of Usability Scale (SUS) Results

Questions	Results
*1 - I think that I would like to use this website frequently*	0% (n = 0) - Strongly disagree
	0% (n = 0) - Disagree
	0% (n = 0) - Neutral
	20% (n = 2) - Agree
	80% (n = 8) - Strongly agree
*2 - I found the website unnecessarily complex.*	70% (n = 7) - Strongly disagree
	30% (n = 3) - Disagree
	0% (n = 0) - Neutral
	0% (n = 0) - Agree
	0% (n = 0) - Strongly agree
*3 - I thought the website was easy to use.*	0% (n = 0) - Strongly disagree
	0% (n = 0) - Disagree
	10% (n = 1) - Neutral
	10% (n = 1) - Agree
	80% (n = 8) - Strongly agree
*4 - I think that I would need the support of a technical person to be able to use this website.*	100% (n = 10) - Strongly disagree
	0% (n = 0) - Disagree
	0% (n = 0) - Neutral
	0% (n = 0) - Agree
	0% (n = 0) - Strongly agree
*5 - I found the various functions in this website were well integrated.*	0% (n = 0) - Strongly disagree
	0% (n = 0) - Disagree
	0% (n = 0) - Neutral
	30% (n = 3) - Agree
	70% (n = 7) - Strongly agree
*6 - I thought there was too much inconsistency in this website.*	100% (n = 10) - Strongly disagree
	0% (n = 0) - Disagree
	0% (n = 0) - Neutral
	0% (n = 0) - Agree
	0% (n = 0) - Strongly agree
*7 - I would imagine that most people would learn to use this website very quickly.*	0% (n = 0) - Strongly disagree
	0% (n = 0) - Disagree
	10% (n = 1) - Neutral
	20% (n = 2) - Agree
	70% (n = 7) - Strongly agree
*8 - I found the website very cumbersome to use.*	80% (n = 8) - Strongly disagree
	20% (n = 2) - Disagree
	0% (n = 0) - Neutral
	0% (n = 0) - Agree
	0% (n = 0) - Strongly agree
*9 - I felt very confident using the website.*	100% (n = 10) - Strongly disagree
	0% (n = 0) - Disagree
	0% (n = 0) - Neutral
	0% (n = 0) - Agree
	0% (n = 0) - Strongly agree
*10 - I needed to learn a lot of things before I could get going with this website.*	80% (n = 8) - Strongly disagree
	10% (n = 1) - Disagree
	0% (n = 0) - Neutral
	0% (n = 0) - Agree
	10% (n = 1) - Strongly agree
*COMMENTS:*	• 3 partners commented that the resources were good • 3 partners commented that the website design was aesthetically pleasing (soothing colours, nice theme, peaceful) • 1 partner commented that they appreciated the different languages and felt that it made the website more inclusive • 1 partner suggested including resources for what to do after a loved one passes

(Brooke, 1996).

### Qualitative Findings

Themes were grouped under three categories: (a) perceptions of the VCoP, (b) recommendations for the VCoP, and (c) experiences in co-designing the VCoP. Quotes were labelled with the study ID of research partners (i.e., RP-XX). See Table 4 for the list of categories and themes.

**Table 4.** Table of Themes

Category	Themes
Perceptions of the VCoP	a) Offers helpful and practical information for dementia care b) Discussion forums are perceived as convenient and promoting sharing
Recommendations to improve the VCoP	a) Ensure information is accessible for caregivers with different needs b) Provide activities that are tailored to life stories c) Uphold normalcy
Experiences in Co-designing the VCoP	a) Felt included and supported during the workshops b) Ideas shared were reflected in the VCoP

### Perceptions of the VCoP

#### Offers Helpful and Practical Information for Dementia Care

Research partners perceived that content included on the website such as how to deliver Namaste Care, effective communication strategies, and pain management for people living with dementia were helpful for caregivers. One research partner shared that: “Having something that is specific and practical resources, like here is an idea of Namaste Care for activities. Not just for the person, but so that you do have an easier time offering the care. I think that is very big deal as well” (RP-09). They reported that practical information was provided that was intuitive and easy to retain when caregiving. “I learned something and whatever I learned was very practical. It was not something you had to work very hard at remembering” (RP-04). A research partner who was a former caregiver commented on how this information could have helped them with their caregiving journey. “I wish I had this type of help or information or resources when I was going through that with my mom” (RP-10).

### Discussion Forums Are Perceived as Convenient and Promoting Sharing

As part of the VCoP intervention, the idea of discussion forums to promote sharing and learning among caregivers was well appreciated by research partners. Research partners perceived that there has been a need to recognize how busy caregivers might be. Virtual options for caregiver discussions provide privacy and flexibility.

It is a good idea and lots of people would appreciate it because before the pandemic, a common way was having support group and that was in-person, it was really difficult for me and I did attend a few times and that was it. It was so much and everybody is busy. I do mentor caregivers through caregiver organizations now and I met some people that are really busy and also need privacy in talking on the phone in the house. I think this online community offers lots of privacy and flexibility for people.” (RP-02)

Research partners recognized that by offering virtual discussion forums for caregivers we are “opening the doors and becoming something that these people [caregivers] need so much” (RP-05). They reported that caregiving can be an isolating experience and there is a potential to neglect the social and emotional needs that caregivers may have in being able to share among other caregivers going through similar experiences in the caregiving journey. One research partner shared:

We need more places that we can talk to each other. So, I think the discussion forum will be really important for that because you know, I think it’s a really important part of caregiving and it is such a huge part that we really end up neglecting ourselves through the process. Emotionally mostly, but physically too. Emotionally, I think it would be wonderful to have a place to go. (RP-01)

### Recommendations for the VCoP

#### Ensure Information Is Accessible for Caregivers With Different Needs

Most research partners perceived that it was easy to locate information on the VCoP. There was an appropriate amount of text included on the pages and navigating between pages was seamless. One research partner shared that:

It [website] is very easy to navigate, and I wasn’t overwhelmed by text and usually when it’s an information website I tend to get quite bogged down by too much information. This is very easy and very clean, and I can spot what I wanted to read.” (RP-10)

Some research partners commented on the appearance of the website in how it draws caregivers to browse the pages:

It’s a very calming peaceful palette of colors. And sometimes the choice of background is also very calming. Like the lavender and it was wonderful pages and the green and pale yellow, the yellow palettes. It is very soothing for people who are already stressed out.” (RP-10)

Despite the positive feedback, research partners perceived that the VCoP needs to provide accessible information in terms of resources and videos in different languages. This would ensure that access to information is equitable and understood by all: “I wish this [video on caregiving] could be translated in French” (RP-04). Research partners also recognized the need to ensure that terminology used could be understood by caregivers with different levels of education. One research partner suggested defining common terms before describing the process, for example, when completing documents such as a living will.

“Let’s say you are dealing with somebody with minimal education. Like if you were going to say, like a will. Well, what is a will? Let’s start with that definition first. So, define what it is, like concisely as possible and simply as possible so then they understand what they are going to be reading about.” (RP-07)

Research partners recognized that some caregivers may have difficulty reading text with smaller fonts and recommended that different and large font sizes be used. “If the fonts are smaller, if you have four different sizes that may not be a problem, but I just thought if someone could be senior, some people may have vision impairment” (RP-02).

### Provide Activities That Are Tailored to Life Stories

In discussing the types of activities that would be delivered through the Namaste Care program, research partners emphasized the need to ensure that caregivers take into account the preferences of people living with dementia. Introducing familiar activities is key to meaningfully engaging with people living with dementia.

I remember years ago that there was a woman in the community, and she was looking after her mother. And I would remember she would often say, join their journey. That always stuck in my mind. And it really helped me to focus on what my mother really liked. What was her life like. I am not going to introduce her to puzzles. She’s never done puzzles before. I am not going to introduce her to playing cards, she doesn’t play cards. So, I would focus on things, like what is it that she wants to do. (RP-08)

In tailoring activities, research partners discussed the need to recognize the cultural identities of people living with dementia and honouring different stages of their life. This was seen as important in reaching people living with dementia and fostering stronger connections. One research partner shared playing songs from her mother’s youth:

I would Google some songs from her childhood and her teenage years because French- Canadians know a lot of songs, traditional songs. And it was like for me, I was like listening to one of your videos and when I would see my mom clap her hands, I knew that she was happy that makes me feel good too.” (RP-04)

### Uphold Normalcy

Research partners perceived the need to create a sense of normalcy for people living with dementia and emphasized that this should be at the forefront of the VCoP including Namaste Care activities. This was an important priority reported by caregivers as dementia has led to many changes in routine life already. Encouraging productivity among people living with dementia was seen as benefiting both caregivers and people living with dementia.

People with dementia should feel productive so dad is still physically capable. I will ask him to help me. To engage him with whatever the activity is. I had to move a chair and I could lift the whole thing, but it was big enough so, I was like “Dad, can you give me a lift?” (RP-09)

Research partners perceived that normalizing dementia and routines also meant ensuring that people living with dementia can still be part of the community and attend familiar events. One research partner shared the importance of her mother having social outings for her own wellbeing.

I bring her to church she wanted to go. That’s what she wanted to do. And I made sure that was out in the community, going to the stores, you know, even though it was hard, one step to go up. I’d asked for help, but people wanted to talk to her and touch her and engage with her and that was really important for me.” (RP-04)

As Canada is a country with a high level of diversity and immigration, research partners brought up the importance of celebrating cultural holidays so that people living with dementia can find joy in familiar celebrations. One research partner is a strong advocate for the francophone community and ensured that her mother’s long-term care home recognized cultural holidays.

I had to advocate, we gotta celebrate the francophone days as well. I have nothing against St. Patty’s Day [St. Patrick’s Day], but what about the St. Jean Baptiste, what about Francophone day? It’s just that every year I see that they seem to forget.” (RP-08)

### Experiences in Co-Designing the VCoP

#### Felt Included and Supported During the Workshops

With regards to participating in the co-design workshop sessions, research partners reported feeling “very, comfortable... felt very included... and I felt safe to say what I wanted to say” (RP-01). They enjoyed having time for open discussions with the workshop facilitator and perceived that there was still a good amount of structure that allowed partners to accomplish the tasks at hand.

I liked the openness, the open discussion. I liked that it was structured but not so much... Structured in that we kind of kept to the theme, kept to the itinerary... but yet still felt that we could actually converse and have a dialogue.” (RP-06)

Research partners felt comfortable sharing sensitive and personal information among a group of strangers, and the workshops themselves were perceived as having a relaxing conversation.

We all made a comment on how it was so lovely to share under such a... I’m going to use the word calming because I can’t think of another one... such a relaxed atmosphere. No sort of hurry up and get your answers prepared.” (RP-05)

The small group provided an intimate setting for research partners to share their ideas for the VCoP and research partners perceived they all had ample opportunity to share their thoughts. The co-design experience was enjoyable for research partners as they perceived they were helping others by sharing their experiences. “I really love this because it gives me a chance to talk about my experiences as a caregiver and hopefully it will help somebody else” (RP-03). Despite being a small group of research partners, co-learning was still occurring and there was good diversity in terms of care experience and background among the group.

Just sharing my real-life experience, I also learned from others, like what they go through, in different settings, different geographic area, really with the diverse background. It was quite an important learning opportunity for me, you know, everybody’s different.” (RP-02)

### Ideas Shared Were Reflected in the VCoP

At the second workshop session, research partners felt that their ideas and recommendations that were shared at the first workshop session were very well incorporated. “There was a tremendous change and for the best too. Because as a caregiver- if I were to be a caregiver again, and would recheck this website, it would definitely be helpful (RP-04)”. The VCoP was perceived as evolving towards meeting the actual information needs of caregivers. Another research partner shared the appreciation she had for the ideas that were considered and incorporated into the updated version of the VCoP website: “She showed us the website... at the end of our participation and had already taken into account several of the ideas. And that’s a new thing! I haven’t seen that ever happening and I’ve been involved in advocacy for a lot of years now” (RP-05). Research partners were quick to notice when their feedback was incorporated and felt that they were being heard and that their ideas were valuable.

### Integration of Quantitative and Qualitative Findings

The qualitative findings helped explain and contextualize the positive usability and engagement ratings observed in the quantitative data. The VCoP received an overall SUS score of 94.5, indicating excellent usability. This finding was reflected in the qualitative theme “Easy to locate information”, where research partners described the website as intuitive, well organized, and easy to navigate. Participants reported that information could be found quickly without being overwhelmed by excessive text. The calming visual design and clear layout were also identified as features that enhanced accessibility and user experience.

The PPEET results were supported by qualitative findings related to participants' experiences in the co-design process. All research partners reported that they were able to express their views freely, felt heard, and were satisfied with the workshops. These findings aligned with the themes “Felt included and supported during the workshops” and “Enjoyed sharing ideas in a small group”. Participants described the workshops as welcoming, respectful, and conducive to open discussion, which helped explain the high levels of engagement reported in the surveys.

The qualitative findings also provided insight into why participants perceived the VCoP positively. Research partners highlighted the practical and relevant nature of the resources, particularly information related to Namaste Care, pain management, and communication strategies. The discussion forums were viewed as a valuable way to reduce caregiver isolation and promote sharing among caregivers. These findings demonstrated how the VCoP addressed participants' informational, social, and emotional needs.

## Discussion

The favorable SUS scores and positive partner feedback collectively indicate that the VCoP has the potential to be a usable, accessible, and contextually relevant digital platform that supports shared learning and emotional well-being among caregivers (Brooke, 1996). These findings align with existing evidence demonstrating that co-design processes involving people living with dementia and their care partners yield interventions that more accurately reflect user priorities and lived experience (Murfield et al., 2022; Wang et al., 2019). The strong PPEET results further reinforce the credibility of the co-design approach, with research partners consistently reporting that they felt meaningfully engaged, supported throughout the process, and able to observe their contributions reflected in the final VCoP environment (Abelson et al., 2016). This level of engagement is particularly noteworthy given documented challenges in achieving authentic co-design. Prior literature highlights that many projects fall short of meaningful participation, resulting in interventions that are overly complex or insufficiently aligned with the needs of end users (Constantin et al., 2022; Moyle et al., 2025).

By conducting a formal evaluation of both VCoP usability and the perceived quality of the co-design experience, this study addresses critical gaps identified in earlier reviews, particularly the lack of empirical assessment of co-design processes and outcomes (Constantin et al., 2022). Overall, the results suggest that a VCoP represents a feasible and acceptable model for supporting caregivers of people living with dementia. The high SUS score indicates that the platform has the potential to effectively delivers practical, directly applicable strategies—including Namaste Care activities, communication support, and pain management guidance—underscoring the importance of involving individuals with lived experience in the development of digital health interventions.

Several VCoP features were identified as especially valuable. The online discussion forums were perceived as convenient, accessible, and instrumental in mitigating social isolation, consistent with evidence that such forums enhance engagement and facilitate peer support and resource sharing (Daynes-Kearney & Gallagher, 2023). Research partners also emphasized the importance of inclusive, individualized resources, including multilingual materials, plain language content, and adjustable visual formats. These considerations are essential given the demographic profile of caregivers—many of whom are middle-aged or older—as well as age-related increases in sensory and functional limitations (Alzheimer Society of Canada, 2025). The Accessibility for Ontarians with Disabilities Act (AODA) further mandates that digital resources meet accessibility standards, reinforcing the need for compliance in the design of virtual caregiver supports (AODA, 2020).

The growing diversity of individuals living with dementia necessitates the development of culturally responsive caregiving resources (Alzheimer Society of Nova Scotia, 2025; National Academies of Sciences et al., 2021). Racialized caregivers, in particular, experience disproportionately high care burdens and elevated psychological distress (Dilworth-Anderson et al., 2020; Whitney et al., 2023). Despite this, resources often inadequately address their linguistic, cultural, or religious needs. The inclusion of diverse research partners in co-design activities is therefore essential to ensure that interventions are broadly relevant, culturally sensitive, and capable of addressing the specific needs of underrepresented caregiver groups. Additionally, tailoring content to support person-centred care and promote normalcy in the delivery of Namaste Care was identified as crucial for strengthening connections with individuals living with dementia.

### Implications for Practice, Policy, and Education

Findings indicate that a co-designed VCoP has the potential to address persistent gaps in caregiver support by providing accessible education, opportunities for shared learning, and a trusted platform for peer interaction. The small-group co-design structure fostered trust, reciprocity, and open dialogue, facilitating high-quality contributions from research partners. The timely integration of feedback reinforced perceptions of meaningful engagement. Ensuring diversity among research partners is central to producing an inclusive final product that reflects the varied needs of caregivers. The VCoP may serve as a valuable resource for health and social care providers seeking to deliver evidence-based information and reduce caregiver isolation. With appropriate policy support, the VCoP could complement national dementia strategies and enhance formal caregiver assistance programs. Nurses play a key role in facilitating the integration of virtual communities of practice into dementia care by providing caregiver education, moderating discussion forums, and supporting the delivery of evidence-based interventions such as Namaste Care. Findings highlight the importance of nurses adopting a person-centred approach that emphasizes accessibility, cultural responsiveness, and tailoring activities to the life history of individuals living with dementia. Additionally, nurses can leverage VCoPs to reduce caregiver isolation, enhance knowledge translation, and strengthen partnerships with family caregivers across home and long-term care settings.

### Strengths and Limitations

Study strengths include a structured and comprehensive evaluation of the co-design process using the PPEET and individual interviews, as well as multiple workshop sessions that enabled iterative refinement of the platform. Limitations include a small and demographically narrow sample. Although the SUS and PPEET have been previously found to be valid and reliable, we did not do any pretesting of the tools in the current study. Recruitment strategies should be expanded to engage more diverse caregivers, including those from racialized, cultural, and faith-based communities, as well as more male caregivers. Digital literacy challenges and limited access to devices may also restrict VCoP usability. Future research should examine the platform’s effectiveness with larger, more diverse samples and assess longer-term outcomes related to caregiver well-being and Namaste Care implementation.

## Conclusions

The VCoP was perceived as a valuable, user-friendly intervention that provides accessible information, promotes shared learning, and offers practical caregiving resources. These findings underscore the importance of authentic engagement with research partners and highlight the potential of co-designed digital tools to support the complex needs of caregivers of people living with dementia.

## Supplemental Material

Supplemental Material - Building a Virtual Community of Practice for Family and Friend Caregivers of People Living With Dementia: A Mixed-Methods Study Supplemental Material for Building a Virtual Community of Practice for Family and Friend Caregivers of People Living With Dementia: A Mixed-Methods Study by Marie-Lee Yous, Ruthie Zhuang, Nicole Beaudry, Susanne Langdon, Paul Lee, Myrna Norman, Sylvie Sylvestre, Aki Tomizawa, Sharon Kaasalainen, Carrie McAiney in Sage Open Nursing

Supplemental Material - Building a Virtual Community of Practice for Family and Friend Caregivers of People Living With Dementia: A Mixed-Methods Study Supplemental Material for Building a Virtual Community of Practice for Family and Friend Caregivers of People Living With Dementia: A Mixed-Methods Study by Marie-Lee Yous, Ruthie Zhuang, Nicole Beaudry, Susanne Langdon, Paul Lee, Myrna Norman, Sylvie Sylvestre, Aki Tomizawa, Sharon Kaasalainen, Carrie McAiney in Sage Open Nursing

## Data Availability

The data for this research consists of questionnaires, interviews and workshop discussions transcriptions and notes. Raw data cannot be publicly released due to the risk of compromising participant confidentiality.*

## Appendix

### Abbreviations

LTC: Long-term care
PPEET: Patient and Public Engagement Evaluation Tool
SD: Standard deviation
SUS: System Usability Scale
QOL: Quality of Life.
