# Supplementary material for: Building a Virtual Community of Practice for Family and Friend Caregivers of People Living With Dementia: A Mixed-Methods Study
Source: SAGE Open Nurs. 2026 Jul 23;12:23779608261473148. doi: 10.1177/23779608261473148 (PMC13396585; doi:10.1177/23779608261473148)
Supplement: Supplemental Material - Building a Virtual Community of Practice for Family and Friend Caregivers of People Living With Dementia: A Mixed-Methods Study [file sj-pdf-1-son-10.1177_23779608261473148.pdf]

Supplementary file 1. Patient and Public Engagement Evaluation Tool (PPEET) Results

Questions	Results
1. What perspective did you bring to Building the Virtual Community of Practice?	10% (n = 1) - **Person living with dementia/Patient/patient advisor/patient partner**<br>90% (n = 9) - **Family member/caregiver**<br>0% (n = 0) - **Staff member**<br>0% (n = 0) - **Community member**<br>0% (n = 0) - **Community partner**
2. How long have you been working with MY as a partner/family/community advisor?	60% (n = 6) - **Less than 6 months**<br>10% (n = 1) - **6 to 12 months**<br>20% (n = 2) - **1 to 2 years**<br>10% (n = 1) - **3 to 4 years**<br>0% (n = 0) - **5 years or more**
3. I had a clear understanding of the purpose of Building the Virtual Community of Practice	10% (n = 1) - **Strongly disagree**<br>0% (n = 0) - **Disagree**<br>0% (n = 0) - **Neither agree nor disagree**<br>10% (n = 1) - **Agree**<br>80% (n = 8) - **Strongly Agree**<br><br>Mean: 4.5, SD = 1.27
4. The supports I needed to participate were available (e.g., access to technology, respite etc.).	10% (n = 1) - **Strongly disagree**<br>0% (n = 0) - **Disagree**<br>10% (n = 1) - **Neither agree nor disagree**<br>10% (n = 1) - **Agree**<br>70% (n = 7) - **Strongly Agree**<br><br>Mean: 4.3, SD = 1.34
5. I had enough information to contribute to the topic being discussed.	0% (n = 0) - **Strongly disagree**<br>0% (n = 0) - **Disagree**<br>0% (n = 0) - **Neither agree nor disagree**<br>10% (n = 1) - **Agree**<br>90% (n = 9) - **Strongly Agree**<br><br>Mean: 4.9, SD= 0.32
6. What else would you like us to know about how your participation in Building the Virtual	• Website link sent in advance giving them time to review before participating<br>• Hoping to have paper documents about the resident's compulsory yearly assessments in LTC<br>o Would help caregiver + resident be better prepared

Community of Practice was supported?	for meeting w/ resident's nurse if they could review the different kinds of assessments before hand<br>• Appreciated moderator letting them know how much they appreciated their participation in the project<br>• Very well supported by ML<br>• ML very accommodating with dates + times<br>• Felt listened to, rewarding to see changes on website
7. I was able to express my views freely.	0% (n = 0) - **Strongly disagree**<br>0% (n = 0) - **Disagree**<br>0% (n = 0) - **Neither agree nor disagree**<br>0% (n = 0) - **Agree**<br>100% (n = 10) - **Strongly Agree**<br><br>Mean: 5, SD = 0
8. I feel that my views were heard.	0% (n = 0) - **Strongly disagree**<br>0% (n = 0) - **Disagree**<br>0% (n = 0) - **Neither agree nor disagree**<br>0% (n = 0) - **Agree**<br>100% (n = 10) - **Strongly Agree**<br><br>Mean: 5, SD = 0
9. A wide range of views on the topics discussed was shared.	0% (n = 0) - **Strongly disagree**<br>0% (n = 0) - **Disagree**<br>0% (n = 0) - **Neither agree nor disagree**<br>20% (n = 2) - **Agree**<br>80% (n = 8) - **Strongly Agree**<br><br>Mean: 4.8, SD = 0.42
10. The individuals participating in Building a Virtual Community of Practice represented a broad range of perspectives on the topic.	0% (n = 0) - **Strongly disagree**<br>0% (n = 0) - **Disagree**<br>0% (n = 0) - **Neither agree nor disagree**<br>40% (n = 4) - **Agree**<br>60% (n = 6) - **Strongly Agree**<br><br>Mean: 4.6, SD = 0.52
11. What else would you like us to know about how you were able to share your views?	• Website now includes our feedback<br>• Felt very comfortable sharing information<br>• Moderator gave a lot of positive feedback<br>• Enjoyed the whole process, better able to guide others<br>• The moderator helped set a comfortable tone and listed subjects. They were a great listener

	• Felt respected, appreciated ability to provide further feedback in writing
12. I think that Building a Virtual Community of Practice achieved its objectives	0% (n = 0) - **Strongly disagree**<br>0% (n = 0) - **Disagree**<br>0% (n = 0) - **Neither agree nor disagree**<br>20% (n = 2) - **Agree**<br>80% (n = 8) - **Strongly Agree**<br><br>Mean: 4.8, S =0.42
13. I am confident the input provided through this initiative will be used by MY.	0% (n = 0) - **Strongly disagree**<br>0% (n = 0) - **Disagree**<br>0% (n = 0) - **Neither agree nor disagree**<br>30% (n = 3) - **Agree**<br>70% (n = 7) - **Strongly Agree**<br><br>Mean: 4.7, SD = 0.48
14. I think the input provided through this activity will make a difference to the work of the organization.	0% (n = 0) - **Strongly disagree**<br>0% (n = 0) - **Disagree**<br>0% (n = 0) - **Neither agree nor disagree**<br>30% (n = 3) - **Agree**<br>70% (n = 7) - **Strongly Agree**<br><br>Mean: 4.7, SD = 0.48
15. What else would you like us to know about the influence you think Building a Virtual Community of Practice will have.	• Online community = great tool to learn + connect w/ people, helps busy caregivers<br>• Very happy to be part of the community + sharing their views<br>• Diverse opinions were important<br>• Noticed that all of the info they shared was implemented<br>• Availability in other languages will help take it further<br>• Can have the power to aid caregivers with tools + info they will require<br>• Very important to meet others w/ similiar experiences<br>o For sharing info but also to recieve info
16. As a result of my participation in Building a Virtual Community of Practice, I am better informed about caregiver needs and how to support them.	0% (n = 0) - **Strongly disagree**<br>0% (n = 0) - **Disagree**<br>0% (n = 0) - **Neither agree nor disagree**<br>40% (n = 4) - **Agree**<br>60% (n = 6) - **Strongly Agree**<br><br>Mean: 4.6, SD = 0.52

17. Overall, I was satisfied with this engagement initiative.	0% (n = 0) - **Strongly disagree**<br>0% (n = 0) - **Disagree**<br>0% (n = 0) - **Neither agree nor disagree**<br>0% (n = 0) - **Agree**<br>100% (n = 10) - **Strongly Agree**<br><br>Mean: 5, SD = 0
18. This engagement initiative was a good use of my time.	0% (n = 0) - **Strongly disagree**<br>0% (n = 0) - **Disagree**<br>0% (n = 0) - **Neither agree nor disagree**<br>0% (n = 0) - **Agree**<br>100% (n = 10) - **Strongly Agree**<br><br>Mean: 5, SD = 0
19. What were the strengths of Building a Virtual Community of Practice?	• Caregivers can go to the virtual community anytime (helps busy caregivers)<br>• Virtual community allows caregivers to connect to others<br>• Sharing experiences & learning about other's experiences<br>• Forward thinking<br>• Diversity of caregivers<br>• Moderator's communication skills<br>• Resources on website were helpful<br>• Website was easy to read and has a pleasing appearance<br>• Resources available in multiple languages<br>• Connecting with others was stimulating, helpful for self-reflection<br>• Small group was good for ample time to share and discuss<br>• Practice tips and resources<br>• Gentle exchange of ideas<br>• Focus on helping caregivers<br>• Virtual and flexible to people's schedules
20. What could be improved about Building a Virtual Community of Practice?	• Not sure (see how virtual community develops over time)<br>• Include financial planning<br>• More voices of experience<br>• Include people who are no longer caregiving to mentor<br>• Resources about what to do when a loved one has passed<br>• Resources about legal support<br>• Recruitment and retention of participants can be challenging, developing strategies for this is needed<br>• Ice breakers at the start of a VCOP could be helpful to get participants more comfortable<br>• Easy platform for communication

	• French speaking VCOP<br>• More basic info (how to bathe, feed, etc.,)<br>• Grow and expand site and resources
21. What else would you like us to know about your experience Building a Virtual Community of Practice?	• Great experience to learn about other caregivers' experiences and needs<br>• Include information on stress, financial planning, caring for ourselves as caregivers, everything that has to do with dementia<br>• Grateful to be a part of this program/ good experience

SD = Standard Deviation

(Abelson et al., 2016)
